# The Relationship between Social Capital and Quality Management Systems in European Hospitals: A Quantitative Study

**DOI:** 10.1371/journal.pone.0085662

**Published:** 2013-12-31

**Authors:** Antje Hammer, Onyebuchi A. Arah, Maral DerSarkissian, Caroline A. Thompson, Russell Mannion, Cordula Wagner, Oliver Ommen, Rosa Sunol, Holger Pfaff, on behalf of the DUQuE Project Consortium

**Affiliations:** 1 Institute for Medical Sociology, Health Services Research and Rehabilitation Science, Faculty of Human Science and Faculty of Medicine, University of Cologne, Cologne, Germany; 2 Department of Epidemiology, University of California Los Angeles Fielding School of Public Health, Los Angeles, California, United States of America; 3 University of California Los Angeles Center for Health Policy Research, Los Angeles, California, United States of America; 4 School of Social Policy, HSMC, University of Birmingham, Birmingham, United Kingdom; 5 NIVEL, Nederlands instituut voor onderzoek van de gezondheidszorg, Utrecht, The Netherlands; 6 Department of Public and Occupational Health, EMGO Institute for Health and Care Research, VU University Medical Center, Amsterdam, The Netherlands; 7 Avedis Donabedian Research Institute (FAD), Universitat Autònoma de Barcelona, Barcelona, Spain; 8 Red de investigación en servicios de salud en enfermedades crónicas (REDISSEC), Spain; University of Ottawa, Canada

## Abstract

**Background:**

Strategic leadership is an important organizational capability and is essential for quality improvement in hospital settings. Furthermore, the quality of leadership depends crucially on a common set of shared values and mutual trust between hospital management board members. According to the concept of social capital, these are essential requirements for successful cooperation and coordination within groups.

**Objectives:**

We assume that social capital within hospital management boards is an important factor in the development of effective organizational systems for overseeing health care quality. We hypothesized that the degree of social capital within the hospital management board is associated with the effectiveness and maturity of the quality management system in European hospitals.

**Methods:**

We used a mixed-method approach to data collection and measurement in 188 hospitals in 7 European countries. For this analysis, we used responses from hospital managers. To test our hypothesis, we conducted a multilevel linear regression analysis of the association between social capital and the quality management system score at the hospital level, controlling for hospital ownership, teaching status, number of beds, number of board members, organizational culture, and country clustering.

**Results:**

The average social capital score within a hospital management board was 3.3 (standard deviation: 0.5; range: 1-4) and the average hospital score for the quality management index was 19.2 (standard deviation: 4.5; range: 0-27). Higher social capital was associated with higher quality management system scores (regression coefficient: 1.41; standard error: 0.64, p=0.029).

**Conclusion:**

The results suggest that a higher degree of social capital exists in hospitals that exhibit higher maturity in their quality management systems. Although uncontrolled confounding and reverse causation cannot be completely ruled out, our new findings, along with the results of previous research, could have important implications for the work of hospital managers and the design and evaluation of hospital quality management systems.

## Introduction

Improving the quality of health care has become a crucial issue for hospitals in recent years. Hospitals in European countries have enacted a wide range of quality improvement strategies, such as incident reporting systems, evidence-based guidelines, breakthrough projects, audits, and performance indicators. Additionally, there is increasing external pressure on hospitals to provide safe, high quality health care. Therefore, the importance of establishing quality management systems within health care organizations has grown and become an important part of quality improvement in hospitals. 

Within this study we defined quality management systems “as a set of interacting activities, methods and procedures used to monitor, control and improve the quality of care” [1]. The degree of implementation of quality improvement strategies depends on professionals’ compliance with policies and procedures within hospital units or departments [2]. Additionally, strategies will be more effectively implemented given specific objectives, a collective will, and hospital management’s cooperation. Senior management’s actions are particularly influential since they are directly responsible for investing in quality improvement strategies and establishing policies and procedures for the actual implementation [3]. Moreover, hospital managers set the direction of the organization, define the priority of quality within the hospital, and guide their hospitals towards successful quality improvement efforts [4,5]. As such, high quality leadership can be assumed to be an effective organizational capability, which is essential for quality improvement in health care settings [5-7]. This quality of leadership is reflected in shared values and mutual trust – social capital – among the hospital management board members. 

The concept of social capital is increasingly used in health services research and can loosely be defined as the quantity and quality of relations among individuals, whether among societies, communities, organizations or groups [8]. Bourdieu defined individualistic social capital as “the aggregate of the actual or potential resources which are linked to possession of a durable network of more or less institutionalized relationships of acquaintance and recognition” [9]. However, collective social capital – as a feature of social systems – “inheres in the structure of relations between persons and among persons. It is lodged neither in individuals nor in physical implements of production” [10]. Putnam has more recently defined social capital as “features of social organization such as networks, norms and social trust that facilitate coordination and cooperation of mutual benefit” [11]. Putnam identifies two specific types of social capital: bonding (internally focused) social capital and bridging (or externally focused) social capital. Bonding social capital facilitates the formation and cohesion of members within a group. Putnam suggests that bonding social capital contributes to group and organizational performance by creating a cohesive environment, which facilitates the effective and efficient attainment of group goals. According to the concept of social capital, common shared values and mutual trust are essential requirements for successful cooperation and coordination within groups like the hospital management board [12].

Organizations, in general, and hospitals, more specifically, with greater social capital can be characterized as having social relations between their members that are based on trust, mutual understanding, common convictions, and shared values [13]. Previous studies found a strong relationship between social capital within hospitals and hospital performance [14-16]. Others have investigated the role of trusting relationships in health care settings [17]. However, no previous work has explored the relationship between bonding social capital in senior management teams and the maturity of quality management systems in hospitals. For this reason, and considering the aforesaid, we expect that higher levels of social capital within a hospital’s management board lead to better implementation of quality improvement strategies. The purpose of this paper is to determine how well the level of social capital within the hospital management board predicts the maturity of quality management systems in hospitals. We hypothesize that social capital within the hospital board – as perceived by the chief executive officer – is positively associated with the implementation of the quality management systems in European hospitals. 

## Materials and Methods

### Conceptual framework

**Figure 1:**
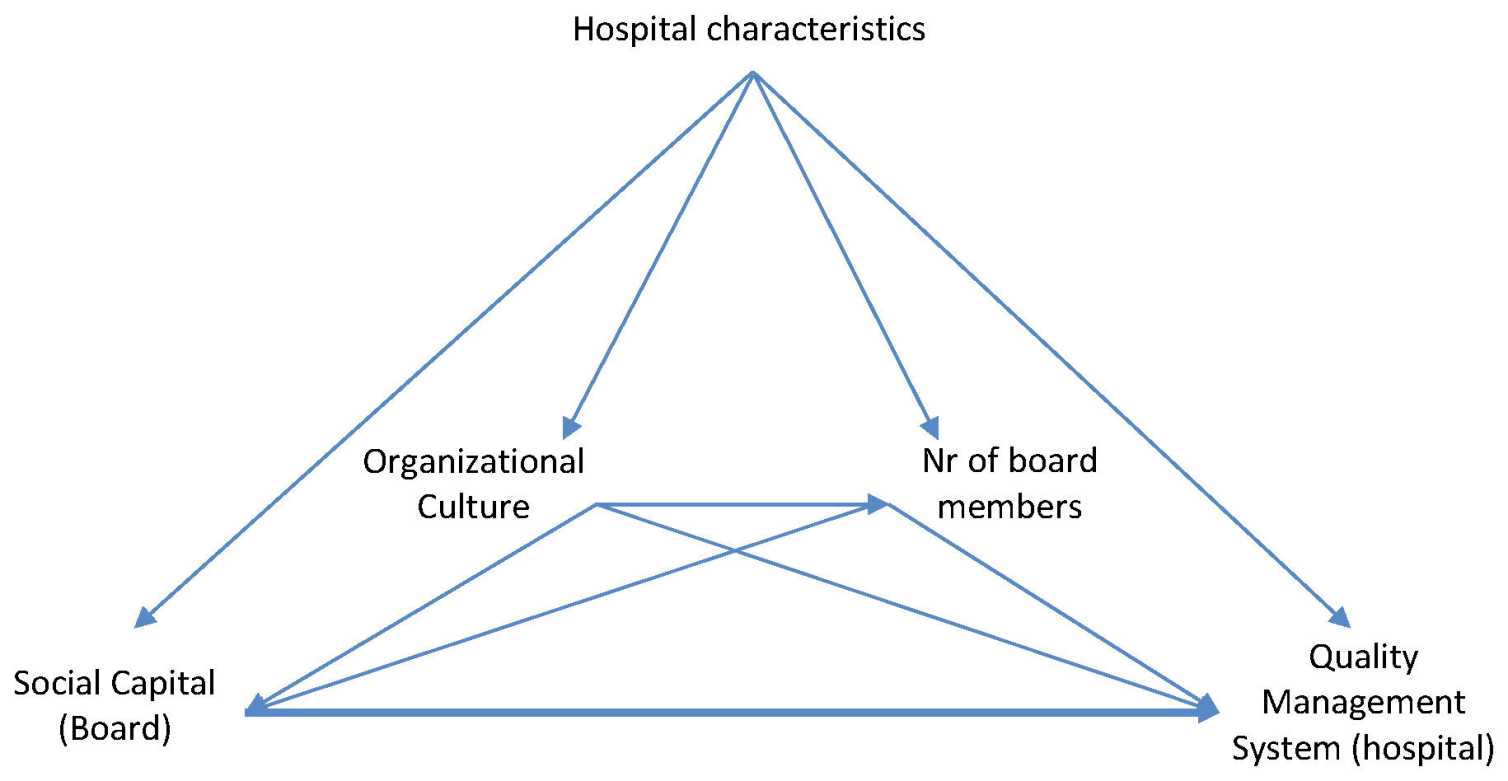
Directed acyclic graph (DAG) of the conceptual framework. The DAG shows the presumed relationship between SC_B_ and QMSI_H_ and the presumed confounding factors organizational culture and number of hospital management board members as well as the hospital structure variables (hospital ownership, teaching status and number of beds).

The purpose of this paper is to examine the relationship between social capital within the hospital management board (SC_B_) and the quality management system at the hospital level (QMSI_H_). Based on previous research, we developed a conceptual model to guide our analysis, which is graphically displayed in Figure 1. To assess the effect of the exposure SC_B_ on the outcome QMSI_H_, we constructed a directed acyclic graph (DAG) as described by Greenland et al. [18]. The analyses described in this paper directly follow the structure of this diagram and the rules it imparts as a DAG.

In accordance with the theoretical background, we presume a positive relationship between SC_B_ and QMSI_H_. In other words, more social capital within the hospital management board is associated with a better quality management system score at the hospital level. Organizational culture (categorical) and number of hospital management board members (continuous), as well as the structural variables hospital ownership (categorical), teaching status (categorical), and number of beds (categorical), are considered as confounding variables. We assume there is no unknown confounding of the relationship between our confounder set and the exposure SC_B_ and the outcome variable QMSI_H_. This assumption allows us to condition on the above mentioned confounders in our analysis, without introducing any further bias after controlling for the confounding factors. 

### Setting, study design and population

This study used data from the project “Deepening our understanding of quality improvement in Europe (DUQuE)” funded by the EU 7th Research Framework Program [19]. The study setting, population, and design are detailed elsewhere [19]. To summarize, the main goal of the DUQuE project was to study the effectiveness of quality improvement systems in European hospitals [19]. Within the project we applied a multi-method approach to data collection and measurements. We approached 210 randomly selected hospitals in Czech Republic, France, Germany, Poland, Portugal, Spain, and Turkey for data collection. The sample was restricted to hospitals with more than 120 beds that handle acute myocardial infection, stroke, hip fracture, and delivery. Data were collected at the hospital, departmental, professional, and patient level. However, data analyzed for this paper consist solely of questionnaire based hospital-level constructs. We used responses from the hospital’s board of trustees, chief executive officer, chief medical officer, highest ranking nurse, and quality manager. Data were collected via web-based questionnaires. The data collection period of the DUQuE-project took place between May 2011 and February 2012. 

### Outcome: Quality Management System

The quality management system index (QMSI_H_) is a newly developed multi-item and multi-dimensional instrument measuring the degree of implementation of quality management systems in hospitals. The development of items within the questionnaire is based on the literature review [1] and previous experiences in this research field [19,20]. Items focus on the managerial aspects of quality management (e.g. quality policies documents, quality monitoring, training of professionals, and formal protocols). The QMSI_H_-index consists of 46 items and nine scales. An overview of the items and scales is provided in Table S1 in Supporting Information. The items on QMSI_H_ were incorporated into the questionnaires for quality managers. Answers were given on 4-point Likert scales ranging from ‘not available’ (1) to ‘fully implemented in (nearly) all relevant inpatient units’ (4) or from ‘strongly disagree’ (1) to ‘strongly agree’ (4). For this study, we used the composite score averaged over the 9 scales. In order to bring the lower bound of the scale down to 0, we subtracted 9 from the total score. Therefore the overall QMSIH-score ranges from 0 to 27 points. Psychometric analyses found satisfactory Cronbach’s alpha (ranging from 0.72 to 0.82) for eight of the nine scales, but a low Cronbach’s alpha (0.48) for the scale ‘analysing feedback & patient experiences’. However, due to the theoretical importance of this scale, it was kept in the index. Results on construct validity testing showed the index being related to recent measures of quality. 

### Exposure: Social Capital

The variable social capital (SC_B_) was designed to measure two key features of social capital: 1) common values and 2) perceived mutual trust in organizations [13]. The development of the items was based on sociological principles and central statements relating to social capital described by Coleman [10], Putnam [11,12], and Fukuyama [21]. The variable consisted of six items (see Table S2 in Supporting Information) and has been used in several previous studies [6,14-16]. The reliability of the social capital scale is high with a Cronbach’s alpha of 0.83 [13]. The SC_B_-items were incorporated into the chief executive officer’s questionnaires to measure their perceptions of social capital within the hospital (management) board. Answers were given on a 4-point Likert scale ranging from ‘strongly disagree’ (1) to ‘strongly agree’ (4). For this study, we used the composite score averaged over the six items so that the total score ranged from 1 to 4 points. 

### Covariates

#### Organizational Culture

Although less evidence exists in health care research than in other fields [20], we hypothesized organizational culture to be associated with SC_B_ and QMSI_H,_ thus potentially confounding the relationship between them. We measured organizational culture using the Competing values framework (CVF). The CVF was initially designed to evaluate indicators of organizational effectiveness [22], and is frequently used in different organizational contexts as a strategic tool when developing supervision and management programs. However, the CVF can also be used to identify the existing culture in organizations, even in health care organizations, and appears to have strong discriminative power [23]. The CVF is reliable with at least three respondents, and was therefore incorporated into the questionnaires of the four top-level managers (the chair of the board of trustees, the chief executive officer, the chief medical officer, and the highest ranking nurse) to indicate organizational culture in each hospital. The framework consists of five dimensions measuring hospital characteristics, leadership, cohesion, emphasis, and rewards. Each of the dimensions consists of four statements, reflecting four different types of organizational culture (clan or group culture; open or developmental culture; hierarchical culture; rational or market culture) [23]. Organizations with a dominant clan culture have a flexible organizational structure, bonded by loyalty and tradition with an emphasis on morale, trust and support for employees and the leader viewed as a mentor. Quality strategies are orientated towards staff empowerment, team building and openness. Organizations with an open culture are similarly characterized by a flexible organizational structure but the key values relate to risk -taking, creativity, innovation and entrepreneurship. Quality strategies are orientated towards developing creative and innovative approaches to continuous quality improvement. In the case of a market culture the organization is bonded by competition and the leader is goal oriented. In the foreground are clear values around being competitive and with an emphasis on winning. Quality strategies are orientated towards improving productivity and enhancing competitiveness. Within organizations with a hierarchical culture the organization is structured so as to support top down bureaucratic control. The focus of the organization is on order, rules and uniformity with an emphasis on predictability with the leader as administrator. Quality strategies are orientated towards developing rules, guidelines and process controls. For each dimension, the respondents distributed a total of 100 points among these four statements according to the level of similarity with their own hospital (0 points = not similar; 100 = points very similar). To determine the hospital culture types for each hospital the average of three respondents (the chief executive officer, the chief medical officer, and the highest ranking nurse) was calculated. The questionnaire of the chair of board of trustees was used only when data from two of the three chosen respondents was available. Within the analyses we used organizational culture as a class variable which represented the dominant culture type for each hospital based on the highest average hospital score. The rational culture type was chosen as reference category for the regression models.

#### Number of Hospital board members

In accordance with previous research in industrial sectors [24,25] we presumed the number of hospital board members impact organizational performance, and thus confound the relationship between SC_B_ and QMSI_H_. For example a higher number of board members implicates greater collective knowledge [26,27] especially when many different professions are represented, which is typical for hospital boards. On the other hand, a bigger hospital board size implies more coordination efforts (e.g. to make appointments), less flexibility (especially in urgent situations), and more difficulty in reaching consensus [24,25,28]. In order to measure the number of hospital board members, the chief executive officers were asked how many board members are formally on their hospital (management) board. For this analysis, the number of hospital board members was included as a continuous variable. 

#### Hospital characteristics

In addition to the aforementioned variables, hospital characteristics including ownership, teaching status, and number of beds were measured with single items. The type of ownership has been considered in similar previous research [29,30], because private for-profit hospitals appear to be more cost-effective, but have a lower quality of care than public and non-for-profit hospitals [31]. Furthermore, we considered the number of beds because larger hospitals have different resources for implementing quality improvement strategies than smaller hospitals. Moreover, we presumed that teaching hospitals would approach patient safety in a different way compared to non-teaching hospitals [7]. Information about hospital ownership, teaching status and number of beds was provided by the hospital coordinator. Regarding hospital ownership, we distinguished between public (1) and non-public (0) ownership. Hospital teaching status was categorized into teaching (1) and non-teaching (0) hospitals. The number of beds was used as a categorical variable, distinguishing between less than 200 beds (1), 200 to 500 beds (2), 501 to 1000 beds (3), and more than 1000 beds (4). Since the data were collected in seven countries, we used appropriate statistical methods to account for clustering of hospitals within countries.

### Statistical Analyses

The dataset used in this analysis contains only hospitals with complete records on all variables used (exposure, confounders, and outcome), and was restricted to hospitals with at least 2, but no more than 25 board members. We first used descriptive statistics to describe the sample data used for this analysis. For categorical variables we calculated frequencies and percentages. For continuous variables, we calculated the minimum, maximum, mean and standard deviation. For the multivariable adjusted analysis aimed at our core research objective, we ran two multilevel linear regression models. Variables controlled for confounding were chosen based upon our conceptual framework (Figure 1). In both models, the sufficient set of confounders beyond the exposure of interest and the outcome were controlled for in order to block all backdoor pathways between the exposure and outcome, and thus adjust for confounding [18]. For the first model, we fit a multivariable linear mixed model with a random intercept by country, and adjusted for fixed effects such as number of beds, teaching status, and ownership at the hospital level. In the second model, we further adjusted for number of hospital board members and the dominant organizational culture type. For both models we calculated estimates and regression coefficients. Moreover, we calculated intra-class correlation coefficients (ICC). The ICC is an indicator of the dependency of observations within the countries [32]. A higher ICC indicates that countries account for a higher portion of the total variance in the outcome, or that within-country observations are more homogenous or similar to each other than between-country observations. 

We conducted all analyses using SAS version 9.3 (SAS Institute Inc. Cary, NC, USA).

### Ethical approval

DUQuE fulfills all the requirements for research projects in the 7^th^ framework of EU DG. Ethical approval was obtained also by the project coordinator at the Bioethics Committee of the Health Department of the Government of Catalonia (Spain). Each country complied with the confidentiality issues according with national legislation or standards of practice available in each country.

## Results

### Descriptive statistics

Overall, 188 out of 210 hospitals (response rate=89.5%) participated in the DUQuE-study. The final dataset used for these analyses contains only hospitals with complete records on all variables (exposure, confounders, and outcome) used in the multilevel linear regression model (N=138).

Characteristics of the hospitals used in this analysis are presented in Table 1. Almost 82.6% (N=114) of the hospitals were public hospitals, while 17.4% (N=24) were non-public hospitals. The sample included 41.3% (N=57) teaching hospitals and 58.7% (N=81) non-teaching hospitals. 10.1% (N=14) of the hospitals had less than 200 beds, 44.2% (N=61) had 200-500 beds, 30.4% (N=42) hospitals had 501-1000 beds and 15.2% (N=21) hospitals had more than 1000 beds. Descriptive statistics of the exposure, outcome and covariate variables are presented in Table 2. The average number of hospital management board members in our sample is 7.7 (SD=4.2). The most frequent dominant hospital culture type is clan culture (33.3%), followed by rational culture (26.0%), open culture (25.3%), and hierarchical culture (15.2%). The mean of the exposure variable SC_B_ is 3.3 (SD =0.5) measured on a scale ranging from 1 to 4, which indicates a high perceived social capital within the hospital management board. Moreover, the average mean of the outcome variable QMSI_H_ is 19.2 (SD =4.5) on a scale ranging from 0 to 27, indicating high implementation of quality management systems in at least one inpatient unit. 

**Table 1:** Characteristics of hospitals used in analysis (N=138).

**Characteristic**	**N**	**%**
Hospitals used in analysis	138	(100)
Czech Republic	25	(18.1)
France	19	(13.7)
Germany	9	(6.5)
Poland	20	(14.4)
Portugal	23	(16.6)
Spain	26	(18.8)
Turkey	16	(11.5)
Teaching Hospitals	57	(41.3)
Public Hospitals	114	(82.6)
Approximate number of beds in hospital		
<200	14	(10.1)
200-500	61	(44.2)
501-1000	42	(30.4)
>1000	21	(15.2)

**Table 2:** Descriptive statistics of exposure, outcome, and covariates (N=138).

	**Min**	**Max**	**Mean**	**(SD)**
Exposure Variable (Scale)				
Social Capital (1-4)	1.3	4.0	3.3	(0.5)
Outcome Variable (Scale)				
QMS-Index (0-27)	9.3	26.8	19.2	(4.5)
Covariates				
Number of Board Members (Continuous)	2.0	25.0	7.7	(4.2)
Organizational Culture, N (%)**^*1*^**				
*Clan*			46	(33.3)
*Open*			35	(25.3)
*Hierarchy*			21	(15.2)
*Rational*			36	(26.0)

^1^ These are the number of hospitals assigned to each organizational type (based on dominant culture type). Of the hospitals used in this analysis, 4 are missing in the organizational culture variable as there was a tie in the mean scores for the culture types (i.e., no clear dominant type).

### Multilevel regression analyses

Results from our multilevel linear models are shown in Table 3. The estimate for SC_B_ in the first model was 1.42 (standard error=0.62, p=0.024). Thus, after adjusting for the hospital characteristics ownership, teaching status, and number of beds, SC_B_ proved to have a positive association with QMSI_H_. We found no associations for hospital characteristics. In the second model, inclusion of the additional hypothesized confounders marginally changed the results (estimate=1.41, standard error=0.64, p=0.029). However, neither organizational culture nor the number of hospital board members appeared to be directly associated with QMSI_H_, conditional on SC_B_. Again, we found no associations for hospital characteristics. The ICC for the first model was 0.191. It became 0.203 in the second model, indicating that 20.3% of the total outcome variance was due to between country variations.

**Table 3:** Regression coefficient estimates (standard errors) from random-intercept linear mixed models for the effect of hospital-level social capital on quality management systems index (QMSI).

	**Model 1 (N=138)^*1*^**		**Model 2 (N=138)^*2*^**	
	*b* (SE)	P-value	*b* (SE)	P-value
Social Capital	1.42 (0.62)	0.0235	1.41 (0.64)	0.0294
Number of Board Members	--	--	-0.08 (0.12)	0.5214
Organizational Culture Type				
*Clan*	--	--	-0.45 (1.04)	0.6671
*Open*	--	--	0.25 (1.13)	0.8248
*Hierarchy*	--	--	0.05 (1.28)	0.9683
*Rational*	--		(ref)	
Number of beds				
*<200*	1.48 (1.65)	0.3708	1.38 (1.72)	0.4242
*200-500*	-0.20 (1.19)	0.8679	-0.20 (1.26)	0.8728
*501-1000*	0.35 (1.24)	0.7748	0.38 (1.27)	0.7625
*>1000*	(ref)		(ref)	
*Public hospital*	0.17 (1.11)	0.8785	0.22 (1.13)	0.8496
*Teaching hospital*	-0.65 (1.24)	0.6025	-0.47 (1.29)	0.7142
ICC	0.191		0.203	

^1^ Multivariable linear mixed model adjusted for fixed effects at the hospital level (number of beds, teaching status, and ownership).

^2^ Additionally adjusted for number of board members (continuous), and organizational culture (classified).

## Discussion

### Main findings

The results of the multilevel linear regression analysis indicated a strong correlation between the exposure SC_B_ and the outcome variable QMSI_H_. More social capital within the hospital management board had a positive effect on the maturity of quality management systems in hospitals. After adjusting for number of hospital board members, organizational culture types, and hospitals characteristics, a one-unit increase in SC_B_ was associated with a 1.41 unit increase in QMSI_H_. The ICC in both models indicated a high variance between the countries. Furthermore, our results suggested that hospital characteristics, organizational culture and the number of hospital board members were not associated with QMSI_H_ when controlling for SC_B_, though the DAG we used assumed that these variables confounded the relationship between SC_B_ and QMSI_H_. Despite this latter finding we may not rule out the possibility of a relationship between these variables and QMSI_H_ however, because such a relationship may be primarily indirect, i.e., through the effect of SC_B_. 

### Strengths and limitations

The results presented are based on a cross-national, multi-method approach to data collection and measurements, which allows for the analysis of factors influencing the implementation of quality improvement strategies at the hospital level across countries. Within this study, we used validated scales as much as possible. Moreover, we used different questionnaires for the independent variable social capital and for the dependent variable quality management system, in order to decrease the risk of common method bias [33].

However, the findings of our analysis are limited in some respects. First, the cross-sectional design of this study does not allow us to draw causal conclusions. In order to do so, we will have to study the nature of this relationship further using longitudinal studies. Second, this analysis is based on data collection across seven countries. The final sample composition and different acceptance rates in each country could have compromised representativeness of the sample. For instance, hospitals in some countries such as Germany and France appear underrepresented in the final sample and might cause selection bias. To account for clustering of hospitals within the countries, we used random intercept by country in our multivariable linear mixed model regression. Moreover, we did not (and, per study agreement, were not allowed to) compare countries in our analyses. Third, the items on social capital and quality management system were only included in the questionnaires of the chief executive officers and the quality managers. The results must therefore be interpreted considering that the data represent the chief executive officer’s perceptions of social capital within the hospital (management) board, and the quality manager’s perceptions of the quality management system at the hospital level. Therefore these findings cannot be generalized without reservations. Nevertheless, the use of key informants, such as members of the hospital management, is common in previous studies because they are presumed to have a comprehensive knowledge of their organizations [34]. Additionally, the results of this study are based on relatively high response rates (about 90% of the expected in the professional questionnaires) in the recruited hospitals. However, due to missing values in the set of variables (namely, exposure, confounders, and outcome variables) used in the multilevel linear regression model, our results are restricted to 138 out of 188 hospitals. In relation to this, the effect of sample size needs to be considered with regard to the precision of our regression model. Since we used random intercept by country in our multivariable linear mixed models, the limited country-level sample size may also be a possible explanation for our inability to detect a relationship between the confounders (especially for organizational culture and the number of hospital board members) and QMSI_H,_ when controlling for SC_B_. Finally, based on our conceptual model, we considered all possible confounders and presumed no unknown confounding of the relationship between our confounder set and the exposure variable SC_B_ and the outcome variable QMSI_H_. However, uncontrolled confounding and reverse causation cannot be completely ruled out and should be considered when interpreting the results. In addition, the presented analysis does not allow further conclusions on the impact of hospital characteristics or organizational culture types nor the number of hospital board members on the exposure variable SC_B_.

## Conclusion

The results suggest that social capital in hospital (management) boards positively influences quality management systems in European hospitals. For practical application, the results indicate a potential advantage of strengthening the existing social capital in hospital management boards, perhaps by building trust through personal development courses or further education on teamwork within the hospital management board. However, additional research is required to further examine the relationship between social capital and quality management systems. Moreover, future studies should investigate whether there are specific hospital characteristics or other contextual factors which influence social capital in order to determine concrete areas for potential improvements to social capital and quality management systems. Additional studies should be conducted, particularly longitudinal studies that may reveal causal relationships. Moreover, stratified, qualitative follow-up studies might be necessary to analyze the influence of social capital in hospital management, and in developing strategies for an effective quality management system.

## Supporting Information

Table S1 **Scales and items of the quality management systems index (QMSI_H_).**
(DOCX)

Table S2 **Items of the social capital scale (SC_B_).**
(DOCX)

## References

[1] Wagner C, Bakker DH de, Groenewegen PP (1999) A measuring instrument for evaluation of quality systems. Int J Qual Health Care 11 (2): 119–130. doi:10.1093/intqhc/11.2.119. PubMed: 10442842. 10442842

[2] Flin R, Mearns K, O'Connor P, Bryden R (2000) Measuring safety climate: identifying the common features. Saf Sci 34: 177–192. doi:10.1016/S0925-7535(00)00012-6.

[3] Zohar D, Tenne-Gazit O (2008) Transformational leadership and group interaction as climate antecedents: A social network analysis. J Appl Psychol 93 (4): 744–757. Available: WOS:000257680700003 18642981 10.1037/0021-9010.93.4.744

[4] Flin R (2007) Measuring safety culture in healthcare: A case for accurate diagnosis. Saf Sci 45 (6): 653–667. doi:10.1016/j.ssci.2007.04.003.

[5] Glickman SW, Baggett KA, Krubert CA, Peterson ED, Schulman KA (2007) Promoting quality: the health-care organization from a management perspective. Int J Qual Health Care 19 (6): 341–348. doi:10.1093/intqhc/mzm047. PubMed: 17947386. 17947386

[6] Hammer A, Ommen O, Röttger J, Pfaff H (2012) The relationship between transformational leadership and social capital in hospitals - a survey of medical directors of all German hospitals. J Public Health Manag Pract 18 (2): 175–180. doi:10.1097/PHH.0b013e31823dea94. PubMed: 22286287. 22286287

[7] McFadden KL, Henagan SC, Gowen III (2009) The patient safety chain: Transformational leadership's effect on patient safety culture, initiatives, and outcomes. JOM 27 (5): 390–404. Available: WOS:000269579900005

[8] Mannion R, Davies H (2005) Taking stock of social capital in the production of health care. J Health Serv Res Policy 10 (3): 129–130. doi:10.1258/1355819054339013. PubMed: 16053587. 16053587

[9] Bourdieu P (1986) The forms of capital. In: Richardson JG Handbook of theory and research for the sociology of education. Connecticut: Greenwood Press pp. 241–258.

[10] Coleman JS (1990) Social capital. Foundations of social theory. Cambridge: Havard Univ. Press: 300–321.

[11] Putnam RD (1995) Bowling alone: America's declining social capital. Journal of Democracy 6: 65–78. doi:10.1353/jod.1995.0070.

[12] Putnam R ( (spring)2001). Social Capital Measurement and Consequences isuma (Spring): 41–51.

[13] Pfaff H, Badura B, Pühlhofer F, Siewerts D (2005) Das Sozialkapital der Krankenhäuser - wie es gemessen und gestärkt werden kann. In: Badura B Schellschmidt H Vetter C Fehlzeiten-Report 2004 : Gesundheitsmanagement in Krankenhäusern und Pflegeeinrichtungen; Zahlen, Daten, Analysen aus allen Branchen der Wirtschaft. Berlin: Springer pp. 81–109.

[14] Gloede TD, Hammer A, Ommen O, Ernstmann N, Pfaff H (2013) Is social capital as perceived by the medical director associated with coordination among hospital staff? A nationwide survey in German hospitals. J Interprof Care 27 (2): 171–176. Available: http://www.ncbi.nlm.nih.gov/pubmed/23016540. PubMed: 23016540. 23016540 10.3109/13561820.2012.724125

[15] Ernstmann N, Ommen O, Driller E, Kowalski C, Neumann M et al. (2009) Social capital and risk management in nursing. J Nurs Care Qual 24 (4): 340–347. doi:10.1097/NCQ.0b013e3181b14ba5. PubMed: 19755881. 19755881

[16] Ernstmann N, Driller E, Kowalski C, Karbach U, Jung J et al. (2012) Social capital and quality emphasis: A cross-sectional multicenter study in German hospitals. International - Journal of Healthcare Management 5 (2): 98–103. doi:10.1179/2047971912Y.0000000007.

[17] Connell NAD, Mannion R (2006) Conceptualisations of trust in the organisational literature: some indicators from a complementary perspective. J Health Organ Manag 20 (5): 417–433. doi:10.1108/14777260610701795. PubMed: 17087403. 17087403

[18] Greenland S, Pearl J, Robins JM (1999) Causal diagrams for epidemiologic research. Epidemiology 10 (1): 37–48. doi:10.1097/00001648-199901000-00008. PubMed: 9888278. 9888278

[19] Groene O, Klazinga N, Wagner C, Arah OA, Thompson A et al. (2010) Investigating organizational quality improvement systems, patient empowerment, organizational culture, professional involvement and the quality of care in European hospitals: the 'Deepening our Understanding of Quality Improvement in Europe (DUQuE)' project. BMC Health Serv Res 10: 281 Available: http://www.biomedcentral.com/1472-6963/10/281. PubMed: 20868470. 20868470 10.1186/1472-6963-10-281 PMC2949856

[20] Shortell SM, O'Brien JL, Carman JM, Foster RW, Hughes EFX et al. (1995) Assessing the impact of continuous quality improvement / total quality management: concept versus implementation. Health Serv Res 30 (2): 377–401. PubMed: 7782222. 7782222 PMC1070069

[21] Fukuyama F (2001) Social capital, civil society and development. Third World Q 22 (1): 7–20. doi:10.1080/713701144.

[22] Quinn RE, Cameron K (1983) Organizational life cycles and shifting criteria of effectiveness: some preliminary evidence. Management Science 29 (1): 33–51. doi:10.1287/mnsc.29.1.33.

[23] Zammuto RF, Krakower JY (1991) Quantitative and qualitative studies of organizational culture. Research in Organizational Change and Development 5: 83–114.

[24] Guest PM (2009) The impact of board size on firm performance: evidence from the UK. European Journal of Finance 15 (4): 385–404. doi:10.1080/13518470802466121.

[25] Lipton M, Lorsch JW (1992) A modest proposal for improved corporate governance. Business Lawyer 48 (1): 59–77.

[26] Dalton DR, Daily CM, Johnson JL, Ellstrand AE (1999) Number of directors and financial performance: a meta-analysis. Academy of Management Journal 42 (6): 674–686. doi:10.2307/256988.

[27] Dalton CM, Dalton DR (2005) Boards of directors: utilizing empirical evidence in developing practical prescriptions. British J Manage 16 (Suppl. S1): S91. doi:10.1111/j.1467-8551.2005.00450.x.

[28] Jensen MC (1993) The modern industrial revolution, exit, and the failure of internal control systems. J Finance 48 (3): 831–880. doi:10.1111/j.1540-6261.1993.tb04022.x.

[29] Farsi M (2004) Changes in hospital quality after conversion in ownership status. Int J Health Care Finance Econ 4 (3): 211–230. doi:10.1023/B:IHFE.0000036047.66483.46. PubMed: 15277779. 15277779

[30] Sloan FAP, Trogdon JGM, Curtis LHP, Schulman KAM (2003) Does the Ownership of the Admitting Hospital Make a Difference? Outcomes and Process of Care of Medicare Beneficiaries Admitted With Acute Myocardial Infarction. Med Care 41 (10): 1193–1205. doi:10.1097/01.MLR.0000088569.50763.15. PubMed: 14515115. 14515115

[31] Devereaux PJ, Heels-Ansdell D, Lacchetti C, Haines T, Burns KE et al. (2004) Payments for care at private for-profit and private not-for-profit hospitals: A systematic review and meta-analysis. CMAJ 170 (12): 1817–1824. doi:10.1503/cmaj.1040722. PubMed: 15184339. 15184339 PMC419772

[32] Twisk JWR (2006) Applied multilevel analysis: a practical guide. Cambridge: Cambridge University Press.

[33] Podsakoff PM, MacKenzie SB, Lee JY, Podsakoff NP (2003) Common method biases in behavioral research: A critical review of the literature and recommended remedies. J Appl Psychol 88 (5): 879–903. doi:10.1037/0021-9010.88.5.879. PubMed: 14516251. 14516251

[34] Rousseau DM (1990) Assessing organizational culture: The case of multiple methods. In: Schneider B Organizational climate and culture. San Fransisco, CA: Jossey-Bass Inc. pp. 153–192.

