# Supplementary material for: The Relationship between Social Capital and Quality Management Systems in European Hospitals: A Quantitative Study
Source: PLoS One. 2013 Dec 31;8(12):e85662. doi: 10.1371/journal.pone.0085662 (PMC3877377; doi:10.1371/journal.pone.0085662)
Supplement: Table S1 — Scales and items of the quality management systems index (QMSIH). (DOCX) [file pone.0085662.s001.docx]

Table S1: Scales and items of the quality management systems index (QMSI_H_)

| **Scales and Items** |
| --- |
| ***Quality policy documents*** |
| 1. Written description of a formally agreed quality policy |
| 1. Quality improvement plan at hospital level (translation of the quality objectives into concrete activities and measures designed to realize the quality policy) |
| 1. Balanced score card (an overview of key quality measures focusing on clinical outcomes, finances, human resources, patient satisfaction) |
| ***Quality monitoring by the board*** |
| 1. The hospital (management) board makes it clear what is expected from care professionals in regards to quality improvement. |
| 1. The hospital (management) board has established formal roles for quality leadership (visible in organizational chart). |
| 1. The hospital (management) board assesses on an annual or bi-annual basis whether care professionals comply with day-to-day patient safety procedures. |
| 1. The hospital (management) board knows and uses performance data for quality improvement. |
| 1. The hospital (management) board monitors the execution of quality improvement plans. |
| ***Training of professionals*** |
| 1. Care professionals are trained by the organization to do their job |
| 1. Care professionals are trained in teamwork |
| 1. Middle management is trained in quality improvement methods |
| 1. Care professionals are trained in quality improvement methods |
| 1. Care professionals are trained in patient safety procedures |
| 1. Care professionals follow at least one training session a year to further develop their professional expertise |
| 1. Care professionals receive information back on the results of their treatment of patients |
| 1. Care professionals are encouraged to report incidents and adverse events |
| 1. Care professional licenses are reviewed by a regulatory body |
| ***Formal protocols for infection control*** |
| 1. Up-to-date hospital protocol for use of prophylactic antibiotics |
| 1. Up-to-date hospital protocol for prevention of central line infection |
| 1. Up-to-date hospital protocol for prevention of surgical site infection |
| 1. Up-to-date hospital protocol for prevention of hospital-acquired infections |
| 1. Up-to-date hospital protocol for prevention of ventilator associated pneumonia |
| ***Formal protocols for medication and patient handling*** |
| 1. Up-to-date hospital protocol for medication reconciliation |
| 1. Up-to-date hospital protocol for the handover of patient information to another care unit |
| 1. Up-to-date hospital protocol for the use of medical aids (e.g., crutches, bandages, etc.) |
| 1. Up-to-date hospital protocol for the prevention of medication errors |
| ***Analysing performance of care processes*** |
| 1. Root-cause analysis of incidents (an incident is an unintended event that have cause or could cause harm to a patient) |
| 1. Risk management (a systematic process of identifying, assessing and taking action to prevent or manage clinical events in the care process) |
| 1. Internal audit (all components of the quality system are periodically assessed with regard to appropriate functioning; i.e., whether all procedures are adhered to and are effective) |
| 1. Monitoring the opinions of care professionals (physicians and nurses are periodically asked about their satisfaction with their work, workload, the terms of employment, etc.) |
| 1. Medical/clinical audit (various disciplines work together to assess and improve the results of care delivery) |
| 1. Adverse event reporting and analysis (clinical staff is required to report and analyse all unexpected and preventable harm to patients caused by medical error or flaws in the healthcare system) |
| 1. Systematic patient record review (systematic reviews of patient records are used to determine adverse events and priorities for quality improvement) |
| 1. Development of care pathways/process redesign (all tests and treatments for a specific patient group are efficiently organized to delivery evidenced based care) |
| ***Analysing performance of professionals*** |
| 1. Hospital (management) board “walk rounds” to identify quality problems and issues (management visits work units to discuss quality and safety issues) |
| 1. Monitoring individual physicians’ performance (physicians undergo systematic and documented performance assessments) |
| 1. Monitoring individual nurses’ performance (nurses undergo systematic and documented performance assessments) |
| ***Analysing feedback& patient experiences*** |
| 1. Benchmarking (specific results (indicators) are compared to other hospital (best in class) in order to identify possible improvement) |
| 1. Monitoring the options of patients (patients are periodically requested to give their opinions on the care provided; include surveys on patient views) |
| 1. Complaints analysis (periodical evaluation of complaints is used to implement improvements) |
| ***Evaluating results*** |
| 1. Data used from clinical indicators to evaluate and adjust care processes |
| 1. Data used from complication registration to evaluate and adjust care processes |
| 1. Data used from incident reporting system to evaluate and adjust care processes |
| 1. Data used from interviews / surveys with / among patients to evaluate and adjust care processes |
| 1. Data used from assessment of guideline compliance to evaluate and adjust care processes |
| 1. Data used from results of internal audits to evaluate and adjust care processes |
