# Supplementary material for: The Relationship between Social Capital and Quality Management Systems in European Hospitals: A Quantitative Study
Source: PLoS One. 2013 Dec 31;8(12):e85662. doi: 10.1371/journal.pone.0085662 (PMC3877377; doi:10.1371/journal.pone.0085662)
Supplement: Table S2 — Items of the social capital scale (SCB). (DOCX) [file pone.0085662.s002.docx]

Table S2: Items of the social capital scale (SC_B_)

| **Items** |
| --- |
| ***Within our Hospital (management) Board*…** |
| 1. …there is unity and agreement. |
| 1. …we trust one another. |
| 1. …there is a “we feeling” among Board members. |
| 1. …the work climate is good. |
| 1. …the willingness to help one another is great. |
| 1. …we share many common values. |
